# Association of a trunk endurance–strength composite with lower-extremity dynamic balance in physically active college students: relative roles of hip abductor strength and weight-bearing ankle dorsiflexion distance

**DOI:** 10.3389/fphys.2026.1861322

**Published:** 2026-09-23

**Authors:** Han han, Chen He, Fan kaibin

**Affiliations:** 1 Jiangsu Police Institute, Department of Police Physical Training, Nanjing, China; 2 Ministry of Sports, Jiangsu Health Vocational College, Nanjing, Jiangsu, China; 3 School of Sports Training, Nanjing Sport Institute, Nanjing, China

**Keywords:** ankle dorsiflexion mobility, core-related function, dynamic balance, hip abductor strength, physically active college students, Y-balance test

## Abstract

**Objective:**

To examine the association between a trunk endurance–strength composite and the Y-Balance Test Lower Quarter (YBT-LQ) composite score in physically active college students, and to determine whether this association remained independent after simultaneous adjustment for hip abductor strength and weight-bearing ankle dorsiflexion distance.

**Methods:**

This cross-sectional study included 263 college students majoring in sport-related disciplines or maintaining longstanding exercise habits. The YBT-LQ composite score was the primary outcome, the trunk endurance–strength composite was the primary exposure, and hip abductor strength and weight-bearing ankle dorsiflexion distance were specified as key competing explanatory variables; these continuous variables were standardized before entry into the regression models. Correlation analyses and hierarchical regression models were used to assess independent associations after adjustment for sex, age, body mass index, training exposure, injury history, low back pain, and sport background. Bootstrap single-mediator models were then applied to examine potential statistical pathways.

**Results:**

Bivariate analyses showed that the trunk endurance–strength composite was moderately positively correlated with the YBT-LQ composite score (r = 0.39). Hip abductor strength (r = 0.64) and weight-bearing ankle dorsiflexion distance (r = 0.42) were also positively correlated with the YBT-LQ composite score, with hip abductor strength showing the strongest association. After adjustment for covariates, the trunk endurance–strength composite remained independently associated with the YBT-LQ composite score (β = 0.28, *p =* 0.011). After further simultaneous inclusion of hip abductor strength and weight-bearing ankle dorsiflexion distance, model explanatory power increased from 0.281 to 0.587; hip abductor strength (β = 0.59, p < 0.001) and weight-bearing ankle dorsiflexion distance (β = 0.33, p < 0.001) both remained significantly and positively associated with the outcome, whereas the effect of the trunk endurance–strength composite was attenuated and was no longer significant (β = 0.10, *p =* 0.136). Path analysis showed that when hip abductor strength was specified as the mediator, the indirect effect was significant (0.169, 95% CI 0.077 to 0.270, *p <* 0.001), with a proportion mediated of 0.612, whereas the direct effect was not significant (*p =* 0.112). When weight-bearing ankle dorsiflexion distance was specified as the statistical pathway variable, the indirect effect was not supported (0.017, 95% CI -0.050 to 0.080, *p =* 0.566). No significant interactions were observed for sex or activity background (*p =* 0.411 and 0.666).

**Conclusion:**

In physically active college students, YBT-LQ composite performance was more directly and consistently associated with hip abductor strength and weight-bearing ankle dorsiflexion distance than with the trunk endurance–strength composite. The statistical association between the trunk endurance–strength composite and dynamic balance was largely explained by hip abductor strength.

## 1 Introduction

Core stability has been proposed to support trunk orientation, deceleration, and force transmission during single-leg support, landing, and changes of direction, thereby contributing to lower-extremity kinetic-chain control (Kibler et al., 2006; Zemková and Zapletalová, 2022). The YBT-LQ is a commonly used field-based test of lower-extremity dynamic balance because it is simple to administer and requires integrated single-leg control. In high-demand athletic populations such as National Collegiate Athletic Association Division I athletes, YBT reach asymmetry has been associated with subsequent injury (Smith et al., 2015). Because this evidence was obtained from elite collegiate athletes, it is cited here only to support the sport-related relevance of YBT-LQ performance, rather than as direct injury-risk evidence for the broader physically active college student sample examined in the present study. Accordingly, this study focused on factors associated with YBT-LQ performance rather than on injury prediction. A more clinically useful question is which neuromuscular domains, including trunk-related capacity, hip abductor strength, and weight-bearing ankle dorsiflexion distance, most directly constrain YBT-LQ performance in physically active college students.

Core stability is best regarded as an umbrella, multidimensional construct rather than a single measure of trunk strength, trunk endurance, or muscle activation. It includes trunk strength and endurance, neuromuscular control, postural regulation, and task-specific coordination for controlling the trunk and pelvis during movement (Kibler et al., 2006; Zemková and Zapletalová, 2022). Hip strength and function may contribute to lumbopelvic and pelvic stabilization during single-leg tasks, but in the present study hip abductor strength was treated as a separate proximal lower-extremity factor rather than as part of the core-related composite. The present study did not attempt to measure core stability in its entirety. Instead, we operationalized the core-related exposure as a trunk endurance–strength composite derived from standardized trunk endurance and trunk strength assessments. Therefore, this measure is referred to as the trunk endurance–strength composite throughout the manuscript.

Several task-related factors support examining trunk, hip, and ankle measures within the same analytical framework. Three-dimensional kinematic studies have shown that YBT performance is associated with trunk lean, hip flexion, and coordinated lower-extremity movement patterns (Kang et al., 2015), whereas reduced YBT performance in individuals with low back pain suggests that trunk-related functional deficits may be expressed during single-leg dynamic stability tasks (Hooper et al., 2016). Restricted distal ankle mobility can alter hip and knee kinematics during single-leg tasks, providing a plausible mechanism by which the weight-bearing ankle dorsiflexion distance examined in the present study may be related to dynamic balance (Rabin et al., 2016; da Costa et al., 2021). Previous studies have also reported associations among hip and ankle mobility, trunk muscle endurance, and dynamic balance, and have shown that dynamic balance may be influenced by multiple neuromuscular factors rather than determined by a single anatomical region (Nakagawa and Petersen, 2018; López-Valenciano et al., 2019). In addition, hip abductor strength has been shown to predict YBT performance in adolescent elite female basketball players, supporting the relevance of proximal lower-extremity strength to YBT-LQ performance (Domínguez-Navarro et al., 2022).

Nevertheless, the independent role of trunk-related measures in dynamic balance remains uncertain. A systematic review and meta-analysis suggested that core training can improve dynamic balance overall (Barrio et al., 2022), whereas a recent multidimensional analysis in physically active young men reported that trunk stability and endurance had limited explanatory value for whole-body dynamic balance (De Los Ríos-Calonge et al., 2024). These inconsistent findings highlight an important gap: many studies have examined core-related, hip, or ankle variables separately, rather than comparing their relative and independent contributions within the same model. Samples have also often been restricted to a single sex, a single sport, or symptomatic populations, limiting the generalizability of existing findings to physically active college students. Therefore, it remains unclear whether the trunk endurance–strength composite remains independently associated with YBT-LQ performance after accounting for hip abductor strength and weight-bearing ankle dorsiflexion distance, or whether this association is largely accounted for by these more task-specific hip and ankle factors.

Against this background, we conducted a cross-sectional analysis in physically active college students to examine whether a trunk endurance–strength composite was associated with YBT-LQ composite performance after accounting for hip abductor strength and ankle dorsiflexion range of motion. We hypothesized that the trunk endurance–strength composite, hip abductor strength, and weight-bearing ankle dorsiflexion distance would each be positively associated with YBT-LQ performance, but that the trunk-related association would be attenuated after adjustment for hip and ankle factors.

## 2 Methods

### 2.1 Study design and reporting framework

This study used a cross-sectional observational design to examine the association between a trunk endurance–strength composite and the YBT-LQ composite score in physically active college students, and to further evaluate the independent contributions of hip abductor strength and weight-bearing ankle dorsiflexion distance to this association, as well as their potential competing or mediating roles. The analytical framework was specified *a priori* at three levels. First, we assessed the basic association between the core-related variable and the dynamic balance outcome. Second, after adjustment for covariates, we used hierarchical regression and mixed-effects modeling to test the relative independence of the key explanatory variables. Third, mediation or competing-pathway analyses were conducted to further characterize the statistical association structure among the trunk endurance–strength composite, hip abductor strength, weight-bearing ankle dorsiflexion distance, and dynamic balance. This report was prepared in accordance with the Strengthening the Reporting of Observational Studies in Epidemiology (STROBE) checklist for cross-sectional studies (von Elm et al., 2008).

### 2.2 Study setting and study period

Participants were recruited from students majoring in sport-related disciplines and from college students with longstanding exercise habits. Recruitment and data collection were completed within the predefined study period, and all testing was conducted in training facilities and teaching venues. To minimize the influence of site and organizational variation on test results, all measurements were performed under relatively consistent field conditions using a standardized protocol. Data collection was conducted in batches, with participants entering the testing schedule according to their pre-existing classes or teaching units; accordingly, class membership was considered a clustering structure in the subsequent statistical analyses. Each testing batch was scheduled at broadly comparable times of day to reduce the potential influence of diurnal variation and testing time on functional performance, as functional and sport-related performance may vary across the day (Atkinson and Reilly, 1996).

### 2.3 Participants and eligibility criteria

Participants were recruited by convenience sampling from students majoring in sport-related disciplines and from college students who regularly engaged in physical activity. They were organized by class or sport group and entered the testing protocol on a voluntary basis. Inclusion criteria were current university enrollment, a sustained background of physical activity, and the ability to complete all functional assessments according to the study protocol. Exclusion criteria included acute injury, neurological or vestibular disorders, recent surgery, inability to complete the full testing protocol, and missing key study variables. After recruitment, screening, and on-site testing, participants were included in the final statistical analyses according to the predefined criteria.

An *a priori* sample size calculation was performed using G*Power 3.1 (Faul et al., 2009). Using the R² increase procedure for a fixed-model multiple linear regression, with the incremental explanatory effect of the core composite specified as the primary effect of interest, the required minimum sample size was estimated at approximately 240 participants based on an effect size of f² = 0.033, α = 0.05, statistical power (1−β) = 0.80, one tested predictor, and 11 total predictors. To account for missing data, exclusions, and data quality control during field testing, the recruitment target was modestly increased, and 263 participants were ultimately included in the statistical analyses.

### 2.4 Ethics approval and informed consent

The study protocol was reviewed and approved by the Ethics Committee of Nanjing Sport Institute (approval number: RT-2025-13) before implementation. Before testing, all participants were informed of the study purpose, test content, and related procedures, and provided written informed consent on a fully informed and voluntary basis. Participation was entirely voluntary, and participants were free to withdraw at any time without penalty. All data were anonymized during collection and processing; personal identifying information was not included in the final analytical dataset, and all materials were used solely for the purposes of this study and managed in accordance with the study protocol.

### 2.5 Study variables

The analytical role of each variable was specified *a priori*. The primary outcome was the YBT-LQ composite score, which represented overall lower-extremity dynamic balance performance. The primary exposure was the trunk endurance–strength composite, which was derived from trunk endurance and trunk strength assessments rather than from a comprehensive multidimensional assessment of core stability. Hip abductor strength and weight-bearing ankle dorsiflexion distance were treated as key competing explanatory variables to examine whether the association between the trunk endurance–strength composite and YBT-LQ performance remained independent after accounting for proximal lower-extremity strength and distal joint mobility.

Potential confounders were selected *a priori* in accordance with the study objectives and included sex, activity group, program track, age, body mass index, weekly training hours, years of training, musculoskeletal injury in the past 12 months, and low back pain in the past 3 months. Sex, activity group, and program track were used to characterize participants’ demographic and sport-related background; age and body mass index reflected basic physical characteristics; weekly training hours and years of training represented training exposure; and recent injury and low back pain were included to control for the potential influence of musculoskeletal health status on functional performance. Because participants entered the testing protocol according to pre-existing classes or teaching units, class membership was specified as the clustering variable and was treated as a random intercept or clustering unit in subsequent models to account for class-level non-independence.

In exploratory analyses, sex × core and activity_group × core interaction terms were additionally constructed to assess whether the association between core stability and dynamic balance differed by sex or activity background. In sensitivity analyses, a pre-specified outlier indicator was used to identify observations with extreme values in the primary analytical variables. Observations were flagged as outliers if any primary continuous variable, including the YBT-LQ composite score, trunk endurance–strength composite, hip abductor strength, or weight-bearing ankle dorsiflexion distance, had an absolute standardized value greater than 3.0. This indicator was used only for sensitivity analyses, including the outlier-excluded model, and did not affect the primary complete-case analysis. In addition to the primary analysis using core_composite_z as the core-related indicator, an alternative core variable was derived from the principal component extracted from the original core function measures to generate a PCA-derived core factor and to examine the consistency of model results across different representations of core function. The roles of all variables and their hierarchical relationships within the analytical framework were specified *a priori* in the study protocol.

### 2.6 Measurement procedures and testing protocol

#### 2.6.1 Overall testing protocol

All measurements were conducted under uniform field conditions according to a predefined protocol. Before testing, participants received standardized instructions and completed a routine warm-up and the necessary familiarization procedures. All assessments were performed in a fixed order, and bilateral tests followed a consistent side-testing sequence that did not vary by sex, activity background, or sport orientation. Adequate rest periods were provided between tests to minimize the influence of cumulative fatigue on subsequent performance. All assessments were performed by evaluators who had received standardized training, and data recording and on-site scoring followed standardized procedures throughout.

#### 2.6.2 Demographic and training background information

Demographic and training background information included age, sex, years of training, and weekly training hours. Musculoskeletal injury in the past 12 months and low back pain in the past 3 months were collected using a standardized procedure and recorded as the injury_12m and low_back_pain_3m variables, respectively. The activity_group variable was used to distinguish participants’ habitual sport participation background, whereas program_track recorded their sport specialization or course track. Both were classified according to predefined grouping criteria and were entered into subsequent models as background variables.

#### 2.6.3 Dynamic balance assessment

Dynamic balance was assessed using the lower-extremity YBT-LQ according to established procedures (Plisky et al., 2009; Shaffer et al., 2013). Participants performed the test barefoot. For each trial, the participant stood on the tested limb at the center of the YBT-LQ testing grid, with the toes positioned behind the starting line, and reached with the contralateral limb in three standardized directions: anterior, posteromedial, and posterolateral. The testing order was fixed for all participants as follows: right anterior, left anterior, right posteromedial, left posteromedial, right posterolateral, and left posterolateral.

Before formal testing, participants received standardized instructions and completed six familiarization trials in each direction for each limb. During formal testing, three valid trials were recorded for each direction on each limb. A trial was considered invalid and repeated if the participant failed to maintain single-leg stance, moved or repositioned the stance foot, touched the floor with the reaching foot before returning to the starting position, kicked the reach indicator, used the reach indicator for support, reported pain, or failed to return the reaching foot to the starting position under control. The stance-limb heel was required to remain in contact with the platform throughout the reach, and heel lift was considered an invalid trial. Arm movements were not restricted, and participants were not required to keep their hands on their hips.

The greatest valid reach distance from the three formal trials was recorded for each direction and each limb. Limb length was measured from the anterior superior iliac spine to the distal end of the medial malleolus and was used to normalize reach distances. The YBT-LQ composite score for each limb was calculated as follows: [(anterior reach distance + posteromedial reach distance + posterolateral reach distance)/(3 × limb length)] × 100%. The right and left limb composite scores were then averaged to represent overall lower-extremity dynamic balance performance, and this bilateral mean composite score served as the primary outcome in subsequent analyses.

#### 2.6.4 Assessment of core-related trunk endurance and strength

Core-related trunk function was assessed using trunk endurance and trunk strength measures. Before testing, participants received standardized instructions and familiarized themselves with the required testing positions. Trunk endurance was assessed using trunk flexion endurance, trunk extension endurance, and bilateral side bridge endurance tests (McGill et al., 1999). For the trunk flexion endurance test, participants maintained a standardized flexed trunk position with the arms crossed over the chest, and the holding time was recorded in seconds. For the trunk extension endurance test, participants maintained the trunk in a horizontal position with the pelvis and lower limbs stabilized, and the holding time was recorded in seconds. For the side bridge endurance test, participants maintained a straight side-bridge position supported by the forearm and feet, and the left and right sides were tested separately. Each endurance test ended when the participant could no longer maintain the required position, showed visible loss of posture, placed additional body support on the floor, or voluntarily stopped because of fatigue or discomfort.

Trunk strength was assessed using trunk flexion, trunk extension, and bilateral trunk rotation strength tests under standardized isometric testing conditions. Participants were positioned and stabilized according to the direction of trunk movement and performed maximal voluntary isometric contractions after standardized instructions and familiarization. Valid peak strength values were recorded for analysis.A strength assessment was considered invalid and repeated if the participant failed to maintain the testing position, showed obvious compensatory movement, or did not complete the contraction according to the evaluator’s instruction. Left and right side-bridge endurance values and left and right trunk rotation strength values were first averaged to obtain bilateral summary values.

Because these measures captured endurance and strength dimensions of trunk function but did not encompass the full multidimensional construct of core stability, the primary exposure was operationalized as a trunk endurance–strength composite. To derive this composite, each raw trunk endurance and trunk strength measure was first standardized using the sample mean and standard deviation. The trunk endurance domain score was calculated as the mean of the standardized trunk flexion endurance, trunk extension endurance, and bilateral side bridge endurance values. The trunk strength domain score was calculated as the mean of the standardized trunk flexion strength, trunk extension strength, and bilateral trunk rotation strength values. The trunk endurance–strength composite was then calculated as the mean of the endurance and strength domain scores, with higher values indicating better trunk-related endurance–strength capacity. The alternative core-related factor used in sensitivity analyses was derived from the same original trunk endurance and strength measures.

#### 2.6.5 Assessment of hip abductor strength

Hip abductor strength was measured using a hand-held dynamometer under standardized isometric testing conditions (Thorborg et al., 2010). Participants were positioned in side-lying with the tested limb uppermost and the pelvis stabilized. The dynamometer was placed against the lateral aspect of the distal thigh, and participants performed a brief maximal voluntary hip abduction contraction against manual resistance after standardized instructions and familiarization. Valid peak force values were recorded, normalized to body mass, and summarized bilaterally for analysis. Adequate rest was provided between side-specific assessments to minimize fatigue. Hip external rotation strength was measured and processed using the same general approach and was used primarily for sample characterization and supplementary analyses.

#### 2.6.6 Assessment of weight-bearing ankle dorsiflexion distance

Weight-bearing ankle dorsiflexion distance was assessed using the weight-bearing lunge test and expressed in centimeters (Powden et al., 2015). This variable represents the maximal toe-to-wall distance achieved during a weight-bearing lunge and should be interpreted as a linear distance-based measure rather than an angular joint-angle measure. Participants stood facing a wall with the tested foot flat on the floor and the knee aligned over the second toe. They were instructed to move the knee toward the wall while keeping the heel in contact with the floor. The foot was progressively moved away from the wall until the maximal distance at which the knee could touch the wall without heel lift was identified. The distance from the tip of the great toe to the wall was measured in centimeters using a ruler or tape measure. Left and right ankles were tested separately, and the mean of the bilateral values was used as the primary weight-bearing ankle dorsiflexion distance measure. Side-to-side asymmetry in weight-bearing ankle dorsiflexion distance was calculated as the absolute difference between the two sides divided by their mean and multiplied by 100. For regression and pathway analyses, the bilateral mean weight-bearing ankle dorsiflexion distance value was standardized before model entry.

#### 2.6.7 Other descriptive functional measures

In addition to the primary analytical variables, several lower-extremity function and motor control measures were collected to characterize the broader functional profile of the sample. Hip external rotation strength was used to describe additional proximal hip function; the single-leg hop, triple hop, crossover hop, and 6-m timed hop were used to describe hop-related lower-extremity functional performance; the Landing Error Scoring System score was used to describe landing movement quality; and simple reaction time was used to describe basic motor response capacity. These variables were used for sample characterization and supplementary interpretation rather than as primary explanatory variables in the main models. All were assessed according to their respective established protocols and summarized using predefined aggregation rules.

### 2.7 Data processing and quality control

Data were processed before modeling according to predefined rules. Recent musculoskeletal injury and low back pain were coded as binary variables. For bilateral measures, side-specific raw values were retained first and then summarized into bilateral mean values and asymmetry indices. Continuous functional measures were standardized using the sample mean and standard deviation before entry into regression and pathway analyses. The trunk endurance–strength composite was derived by first standardizing the original trunk endurance and trunk strength indicators, then calculating endurance and strength domain scores, and finally averaging the two domain scores. Hip abductor strength and weight-bearing ankle dorsiflexion distance were standardized from their corresponding raw continuous measures for multivariable analyses. In the sensitivity analyses, the first principal component was extracted from the predefined trunk endurance and strength indicators and re-standardized to create an alternative trunk-related factor. Pre-specified outlying observations were identified and handled according to the study protocol, and continuous variables were centered when necessary.

Pre-specified outlying observations were identified using the same outlier indicator described above. Specifically, observations with an absolute standardized value greater than 3.0 in any primary continuous analytical variable were flagged. The influence of these observations was evaluated through sensitivity analyses by comparing the primary model with an outlier-excluded model and a robust-estimation model.

All assessments were performed by the same evaluation team under standardized field conditions and according to a predefined protocol. Test instructions, warm-up, familiarization, and test order were kept consistent across batches; necessary recovery periods were provided between tests; and testing was scheduled, whenever possible, at broadly comparable times of day to reduce the effects of learning, fatigue, and testing-time variation. Raw records were checked for consistency after testing, and participants with missing key variables were excluded from the relevant analyses. Outliers, extreme values, and pre-flagged cases were handled according to predefined rules, and their influence on the main results was evaluated through sensitivity analyses. Because participants were tested by class or teaching unit, class_id was treated as the clustering source in the subsequent analyses.

### 2.8 Statistical analysis

Continuous variables were reported as mean ± standard deviation (SD) or median IQR according to their distributional characteristics, and categorical variables were reported as n (%). Sex-stratified group comparisons were conducted using methods appropriate to variable type and distribution: independent-samples t tests for approximately normally distributed continuous variables, Wilcoxon rank-sum tests for skewed continuous variables, and Pearson χ² tests or exact tests, when appropriate, for categorical variables. Bivariate associations among the trunk endurance–strength composite, hip strength, weight-bearing ankle dorsiflexion distance, and YBT-LQ composite score were evaluated using a correlation matrix, with Pearson correlations as the primary method. Spearman correlations were used when the distribution of either variable in a given pair showed marked non-normality, defined as an absolute skewness greater than 1.0 or an absolute excess kurtosis greater than 3.0, or when influential outlying observations were identified, defined as standardized values exceeding ±3.0.

The YBT-LQ composite score was analyzed using a hierarchical modeling strategy. Model 0 was an empty model including only a random intercept for class membership and was used to estimate the intraclass correlation coefficient. Model 1 included sex, age, body mass index, weekly training hours, years of training, musculoskeletal injury in the past 12 months, low back pain in the past 3 months, activity group, and program track. Model 2 added the trunk endurance–strength composite to Model 1. Model 3 further added hip abductor strength and weight-bearing ankle dorsiflexion distance. Model 4 added the interactions of the trunk endurance–strength composite with sex and activity group to Model 3. Linear mixed-effects models were used preferentially; if the random effect approached zero, the model showed a singular fit, or class_id could not be estimated stably, ordinary linear regression was used instead with cluster-robust standard errors at the class_id level. Model results were reported as unstandardized regression coefficients (B) with 95% confidence intervals, together with standardized beta coefficients (β), p values, marginal R², conditional R², and Akaike information criterion; intercept terms were not presented in the main table.

To estimate standardized coefficients, continuous variables were standardized and refitted under equivalent model structures to ensure comparability across predictors measured on different scales.

For exploratory competing-pathway analyses, two separate bootstrap indirect-association models were constructed, with the trunk endurance–strength composite as the exposure and the standardized YBT-LQ composite score as the outcome. Hip abductor strength and weight-bearing ankle dorsiflexion distance were entered separately as statistical pathway variables in two independent models rather than simultaneously in a parallel mediation model. Because the study used a cross-sectional design, these models were used to describe indirect statistical associations rather than to establish temporal or causal mediation. Covariates were specified identically to those in the main models. The number of bootstrap resamples was set *a priori* at 5000, and the indirect association, residual direct association, total association, and proportion statistically accounted for were reported along with their 95% confidence intervals and p values (Preacher and Hayes, 2004). In the exploratory analyses, the core_composite_z × sex and core_composite_z × activity_group interactions were tested *a priori*; if these interaction terms were not statistically significant, the corresponding prediction plots were presented for illustrative purposes only, and all predicted values shown were based on adjusted model estimates rather than raw means. Sensitivity analyses included an outlier-excluded model, a robust-estimation model, and a model replacing core_composite_z with the PCA-derived core factor. Diagnostics for the main models were evaluated using residual distributions, homoscedasticity, and influence plots. Unless otherwise specified, analyses were conducted using a complete-case approach, all tests were two-sided, and statistical significance was set at α = 0.05. All analyses were performed in R, primarily using the lme4, lmerTest, sandwich, clubSandwich, and lmtest packages.

## 3 Results

### 3.1 Participant characteristics

A total of 263 participants were included in the final analyses, comprising 131 women and 132 men. Baseline characteristics are presented in **Table 1**. Sex-stratified comparisons were performed using independent-samples t tests for approximately normally distributed continuous variables, Wilcoxon rank-sum tests for skewed continuous variables, and Pearson χ² or Fisher exact tests for categorical variables, as appropriate. Based on these prespecified tests, men showed higher body mass index, longer weekly training duration, a higher prevalence of musculoskeletal injury in the past 12 months, greater hip strength, higher YBT-LQ composite scores, better hop-test performance, and higher trunk endurance and strength scores. Women showed greater weight-bearing ankle dorsiflexion distance and a higher prevalence of low back pain in the past 3 months. No significant sex differences were observed in age or training years. These baseline differences were considered in the subsequent adjusted models.

**Table 1:** Participant characteristics overall and by sex.

Variable	Overall^1^	Female^1^	Male^1^	p value^2^
Age, years	20.30 [19.60, 21.10]	20.30 [19.80, 21.00]	20.30 [19.55, 21.20]	0.865
Body mass index, kg/m^2^	22.53 ± 1.95	22.00 ± 1.88	23.05 ± 1.87	<0.001
Training volume, hours/week	8.50 [6.20, 11.60]	7.50 [5.50, 10.70]	10.15 [7.35, 12.15]	<0.001
Training experience, years	5.53 ± 1.98	5.32 ± 1.98	5.75 ± 1.95	0.073
Physical activity group				0.004
RegularActive	131 (49.8%)	77 (58.8%)	54 (40.9%)	
SportsMajor	132 (50.2%)	54 (41.2%)	78 (59.1%)	
Sports major				0.004
0	131 (49.8%)	77 (58.8%)	54 (40.9%)	
1	132 (50.2%)	54 (41.2%)	78 (59.1%)	
Program track				0.004
Basketball	26 (9.9%)	8 (6.1%)	18 (13.6%)	
FitnessClub	65 (24.7%)	44 (33.6%)	21 (15.9%)	
IntramuralSports	33 (12.5%)	16 (12.2%)	17 (12.9%)	
PhysicalEducation	55 (20.9%)	28 (21.4%)	27 (20.5%)	
RecreationRunner	33 (12.5%)	17 (13.0%)	16 (12.1%)	
Soccer	25 (9.5%)	6 (4.6%)	19 (14.4%)	
TrackField	26 (9.9%)	12 (9.2%)	14 (10.6%)	
Any musculoskeletal injury in past 12 months	61 (23.2%)	23 (17.6%)	38 (28.8%)	0.031
Low back pain in past 3 months	26 (9.9%)	18 (13.7%)	8 (6.1%)	0.037
Weight-bearing ankle dorsiflexion distance, cm	11.25 ± 0.97	11.46 ± 0.98	11.05 ± 0.92	<0.001
Weight-bearing ankle dorsiflexion distance asymmetry, %	7.08 [3.05, 12.96]	5.96 [2.64, 11.98]	8.07 [3.73, 14.59]	0.020
Hip abductor strength, N/kg	2.38 ± 0.38	2.19 ± 0.31	2.57 ± 0.35	<0.001
Hip external rotator strength, N/kg	1.57 ± 0.26	1.48 ± 0.23	1.66 ± 0.25	<0.001
Y-Balance composite score, %	90.60 ± 2.28	90.19 ± 2.09	91.01 ± 2.40	0.003
Single-hop distance, cm	171.55 [162.90, 185.15]	165.15 [155.20, 170.80]	183.93 [171.78, 193.33]	<0.001
Triple-hop distance, cm	535.15 [493.10, 576.85]	501.95 [473.05, 529.45]	575.65 [544.70, 605.10]	<0.001
Crossover-hop distance, cm	490.15 [461.45, 525.50]	467.20 [443.60, 490.15]	520.33 [490.55, 553.40]	<0.001
Timed 6-m hop, s	2.07 ± 0.13	2.13 ± 0.11	2.02 ± 0.12	<0.001
Landing Error Scoring System (LESS)	4.00 [3.00, 5.00]	4.00 [3.00, 5.00]	4.00 [3.00, 5.00]	0.089
Simple reaction time, ms	278.60 [252.70, 311.10]	281.30 [255.80, 322.70]	276.60 [247.00, 298.30]	0.108
Core endurance z score	0.00 ± 0.94	-0.32 ± 0.82	0.32 ± 0.95	<0.001
Core strength z score	0.00 ± 0.89	-0.39 ± 0.76	0.38 ± 0.85	<0.001
Trunk endurance–strength composite z score	0.00 ± 0.90	-0.36 ± 0.78	0.35 ± 0.88	<0.001

^1^ Median [Q1, Q3]; Mean ± SD; n (%).

^2^ Wilcoxon rank sum test; Welch Two Sample t-test; Pearson's Chi-squared test

Values are presented as mean ± SD, median (IQR), or n (%), as appropriate. Sex-stratified comparisons were conducted using independent-samples t tests for approximately normally distributed continuous variables, Wilcoxon rank-sum tests for skewed continuous variables, and Pearson χ² or Fisher exact tests for categorical variables. Corresponding p values are shown in the table.

### 3.2 Descriptive statistics and bivariate associations

**Table 2** presents the descriptive statistics and bivariate correlations among the trunk endurance–strength composite, hip strength, weight-bearing ankle dorsiflexion distance, and YBT-LQ composite score. The trunk endurance–strength composite was moderately positively correlated with the YBT-LQ composite score (r = 0.39). Hip abductor strength showed the strongest correlation with YBT-LQ performance (r = 0.64), followed by hip external rotation strength (r = 0.63) and weight-bearing ankle dorsiflexion distance (r = 0.42). Trunk endurance, trunk strength, and the trunk endurance–strength composite were highly intercorrelated (r = 0.94–0.99), supporting the use of the composite trunk-related measure in subsequent analyses.

**Table 2:** Descriptive statistics and bivariate correlations among trunk endurance and strength, hip strength, weight-bearing ankle dorsiflexion distance, and YBT-LQ variables.

			Correlations						
No.	Variable	Descriptive statistic	1	2	3	4	5	6	7
1	Core endurance	0.00 ± 0.94	—						
2	Core strength	0.00 ± 0.89	0.94***	—					
3	Trunk endurance–strength composite	0.00 ± 0.90	0.99***	0.98***	—				
4	Hip abductor strength	2.38 ± 0.38	0.46***	0.51***	0.49***	—			
5	Hip external rotation strength	1.57 ± 0.26	0.44***	0.45***	0.45***	0.81***	—		
6	Weight-bearing ankle dorsiflexion distance	11.25 ± 0.97	0.07	0.04	0.05	0.08	0.06	—	
7	YBT-LQ composite	90.60 ± 2.28	0.39***	0.38***	0.39***	0.64***	0.63***	0.42***	—

Values are shown as mean ± SD for approximately normal variables and median [IQR] for non-normal variables.

Cells show correlation coefficients. Pearson correlations are shown by default; † indicates Spearman correlation. ***p < 0.001.

### 3.3 Hierarchical regression models for the YBT-LQ composite score

The empty model showed a weak clustering effect at the class level, with an intraclass correlation coefficient of 0.031. The hierarchical regression results are presented in **Table 3**. In the covariate-only model, the marginal R² was 0.244. After the trunk endurance–strength composite was added, model explanatory power increased to 0.281, and the trunk endurance–strength composite was positively associated with the YBT-LQ composite score (B = 0.70, 95% CI 0.16 to 1.24; standardized β = 0.28, p = 0.011). Training hours per week and injury in the past 12 months were also significant in this model.

**Table 3:** Hierarchical / mixed-effects regression models for YBT-LQ composite score.

Predictor	Model 1	Model 2	Model 3	Model 4
Sex: Male vs Female	0.21 [-0.01, 0.97], p=0.056	0.07 [-0.33, 0.63], p=0.542	-0.15 [-0.72, 0.04], p=0.079	-0.15 [-0.74, 0.05], p=0.086
Age	-0.01 [-0.35, 0.30], p=0.874	-0.02 [-0.38, 0.29], p=0.803	0.01 [-0.20, 0.25], p=0.833	0.01 [-0.20, 0.23], p=0.915
Body mass index	0.04 [-0.07, 0.17], p=0.411	0.05 [-0.04, 0.17], p=0.251	0.02 [-0.10, 0.14], p=0.741	0.02 [-0.10, 0.14], p=0.738
Training hours/week	0.44 [0.11, 0.51], p=0.003	0.37 [0.05, 0.47], p=0.015	0.10 [-0.04, 0.18], p=0.236	0.08 [-0.06, 0.18], p=0.325
Training years	-0.09 [-0.28, 0.07], p=0.243	-0.10 [-0.30, 0.07], p=0.235	-0.02 [-0.16, 0.12], p=0.758	-0.03 [-0.16, 0.10], p=0.666
Injury in past 12 months: Yes vs No	-0.73 [-2.15, -1.19], p<0.001	-0.62 [-2.12, -0.71], p<0.001	-0.19 [-0.97, 0.09], p=0.106	-0.20 [-0.98, 0.06], p=0.085
Low back pain in past 3 months: Yes vs No	-0.39 [-1.77, 0.01], p=0.053	0.01 [-1.09, 1.12], p=0.979	-0.15 [-1.00, 0.33], p=0.327	-0.12 [-0.95, 0.41], p=0.442
Activity group: Sports major vs Regularly active	-0.38 [-2.17, 0.43], p=0.188	-0.45 [-2.18, 0.13], p=0.082	-0.27 [-1.33, 0.08], p=0.083	-0.25 [-1.27, 0.15], p=0.122
Program track	-0.13 [-0.70, 0.13], p=0.174	-0.13 [-0.72, 0.12], p=0.162	-0.01 [-0.65, 0.61], p=0.947	-0.01 [-0.61, 0.58], p=0.965
Trunk endurance–strength composite		0.28 [0.16, 1.24], p=0.011	β = 0.10; 95% CI for B: −0.08 to 0.60; p = 0.136	0.20 [0.18, 0.83], p=0.002
Hip abductor strength			β = 0.59; 95% CI for B: 1.02 to 1.66; p < 0.001	0.59 [1.01, 1.67], p<0.001
Weight-bearing ankle dorsiflexion distance			β = 0.33; 95% CI for B: 0.50 to 1.02; p < 0.001	0.33 [0.51, 1.01], p<0.001
Core × sex				-0.08 [-0.93, 0.38], p=0.411
Trunk endurance–strength composite × activity group				-0.04 [-0.80, 0.51], p=0.666
N	263	263	263	263
Marginal R²	0.244	0.281	0.587	0.591
Conditional R²	0.244	0.281	0.587	0.591
AIC	1135.6	1124.7	982.6	984.1

Model 0 ICC = 0.031.

Cells report standardized regression coefficients (β), 95% confidence intervals for the corresponding unstandardized coefficients (B), and p values. The confidence intervals are not on the standardized β scale. Intercepts are not shown.

Linear mixed-effects models with random intercept for class_id were preferred. When unstable or singular, ordinary linear regression with class_id-clustered robust standard errors was used.

Program track was retained as a fixed effect in all models; category-specific coefficients are not displayed.

Compared with Model 3, Model 4 increased marginal R² from 0.587 to 0.591 and increased AIC from 982.6 to 984.1.

After hip abductor strength and weight-bearing ankle dorsiflexion distance were added, model fit improved markedly, with marginal R² increasing to 0.587 and AIC decreasing to 982.6. Hip abductor strength (B = 1.34, 95% CI 1.02 to 1.66; standardized β = 0.59, p < 0.001) and weight-bearing ankle dorsiflexion distance (B = 0.76, 95% CI 0.50 to 1.02; standardized β = 0.33, p < 0.001) remained positively associated with the YBT-LQ composite score, whereas the trunk endurance–strength composite was attenuated and no longer significant (B = 0.26, 95% CI -0.08 to 0.60; standardized β = 0.10, p = 0.136). **Figure 1** illustrates the standardized effect sizes of the main predictors in the final model.

**Figure 1:**
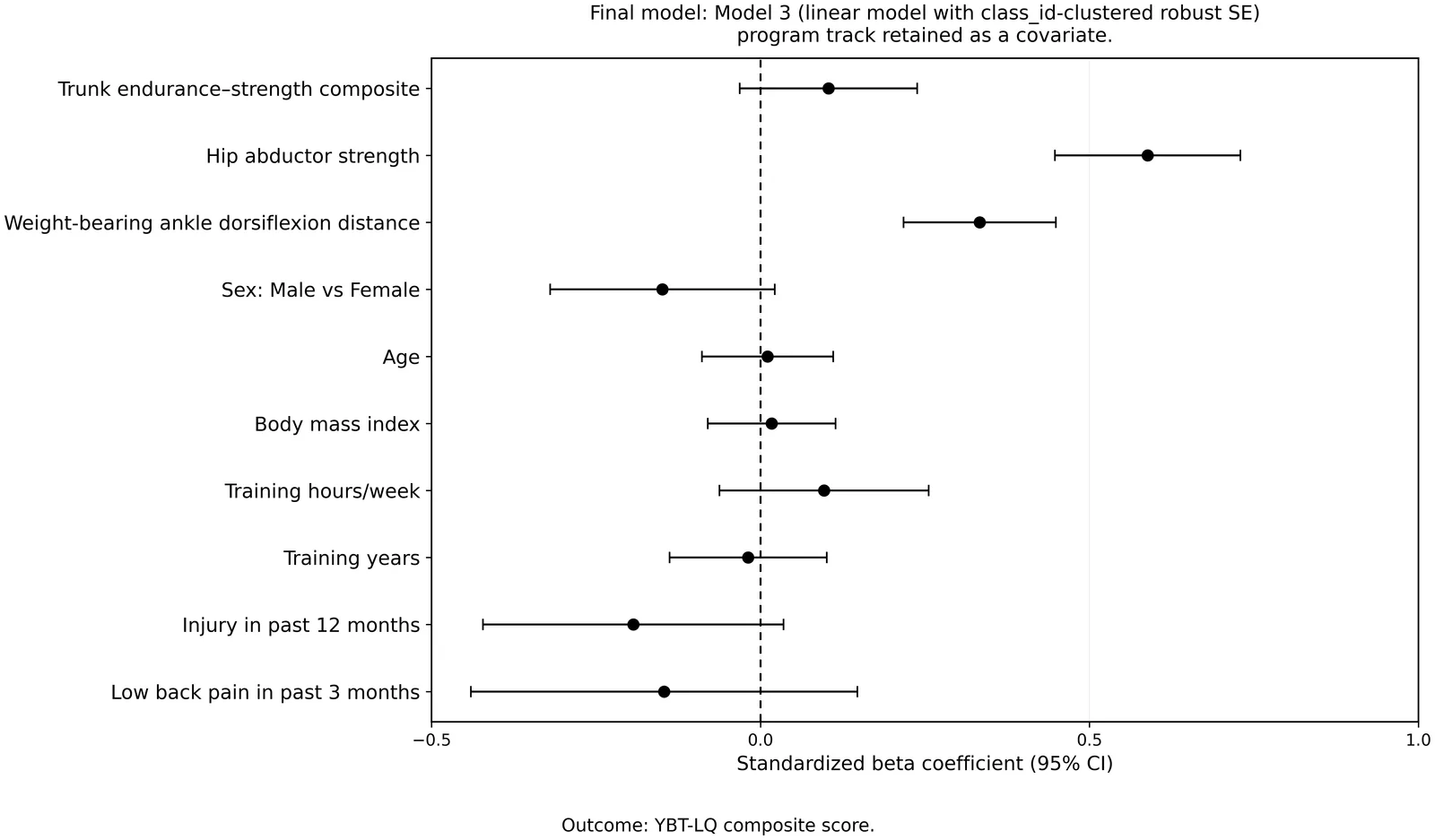
Standardized beta coefficients in the final model predicting YBT-LQ composite score.

Adding the interactions of the trunk endurance–strength composite with sex and activity group produced only a small increase in marginal R² from 0.587 to 0.591 (ΔR² = 0.004) and increased AIC from 982.6 to 984.1 (ΔAIC = +1.5). Neither interaction term was statistically significant: trunk endurance–strength composite × sex (p = 0.411) and trunk endurance–strength composite × activity group (p = 0.666). Therefore, Model 3 was retained as the more parsimonious final main-effects model.

### 3.4 Exploratory competing-pathway analyses

Exploratory competing-pathway analyses were conducted using two independent bootstrap indirect-association models. The first model examined the indirect statistical association between the trunk endurance–strength composite and the YBT-LQ composite score through hip abductor strength, whereas the second model examined the corresponding pathway through weight-bearing ankle dorsiflexion distance. The two pathway variables were not entered simultaneously in a parallel mediation model. Both models were adjusted for the same covariates as the main regression models. Results are presented in **Table 4**, and the two separate model structures are shown in **Figure 2**.

**Table 4:** Bootstrapped indirect-association models of hip abductor strength and weight-bearing ankle dorsiflexion distance in the trunk endurance–strength composite–YBT relationship.

Mediation model	Effect	Estimate	95% CI	P value
Hip abduction mediation model	Indirect effect	0.169	[0.077, 0.270]	<0.001
	Direct effect	0.107	[-0.027, 0.246]	0.112
	Total effect	0.276	[0.105, 0.450]	0.001
	Proportion mediated	0.612	[0.327, 1.186]	0.001
Weight-bearing ankle dorsiflexion distance mediation model	Indirect effect	0.017	[-0.050, 0.080]	0.566
	Direct effect	0.259	[0.107, 0.420]	0.001
	Total effect	0.276	[0.114, 0.454]	0.002
	Proportion mediated	0.062	[-0.306, 0.288]	0.565

N = 263.

Standardized variables were used for the mediation analyses.

Two independent single-mediator bootstrap models were fitted: Trunk endurance–strength composite → Hip abductor strength → YBT-LQ composite, and Trunk endurance–strength composite → Weight-bearing ankle dorsiflexion distance → YBT-LQ

Models were adjusted for sex, age, body mass index, training hours per week, training years, injury in the past 12 months, low back pain in the past 3 months, activity group, and program track.

Bootstrap confidence intervals and p values were based on 5000 resamples.

**Figure 2:**
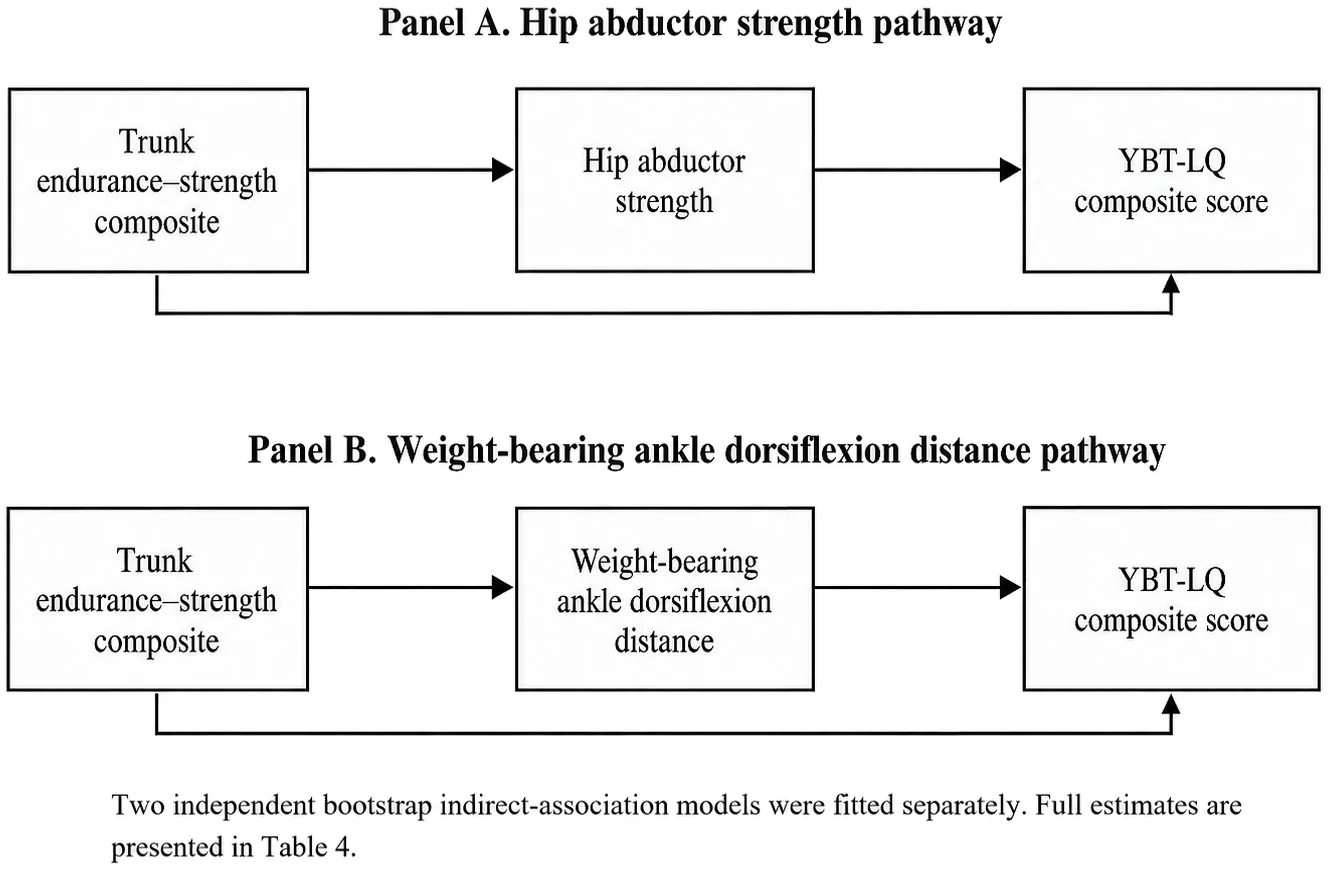
Adjusted competing-pathway models linking the trunk endurance–strength composite to YBT-LQ composite score via hip abductor strength and weight-bearing ankle dorsiflexion distance.

In the hip abductor strength model, the indirect association was significant (0.169, 95% CI 0.077 to 0.270, p < 0.001), whereas the residual direct association was not significant (0.107, 95% CI -0.027 to 0.246, p = 0.112). In contrast, in the weight-bearing ankle dorsiflexion distance model, the indirect association was not statistically significant (0.017, 95% CI -0.050 to 0.080, p = 0.566), and the proportion statistically accounted for was likewise not significant (0.062, 95% CI -0.306 to 0.288, p = 0.565), although the residual direct association (0.259, 95% CI 0.107 to 0.420, p = 0.001) and total association (0.276, 95% CI 0.114 to 0.454, p = 0.002) remained significant. These findings suggest that hip abductor strength statistically accounted for a substantial part of the association between the trunk endurance–strength composite and YBT-LQ performance, whereas weight-bearing ankle dorsiflexion distance did not show a supported indirect pathway.

Overall, based on the bootstrap indirect-effect analyses, the association between the trunk endurance–strength composite and the YBT-LQ composite score was reflected primarily through an indirect pathway via hip abductor strength, whereas the indirect pathway through weight-bearing ankle dorsiflexion distance was not statistically supported.

### 3.5 Exploratory interaction and supplementary analyses

The exploratory interaction analyses did not support significant moderation by sex or activity group. Therefore, **Figure 3** should be interpreted as an illustrative display of adjusted predicted trends rather than as confirmatory evidence of interaction effects.

**Figure 3:**
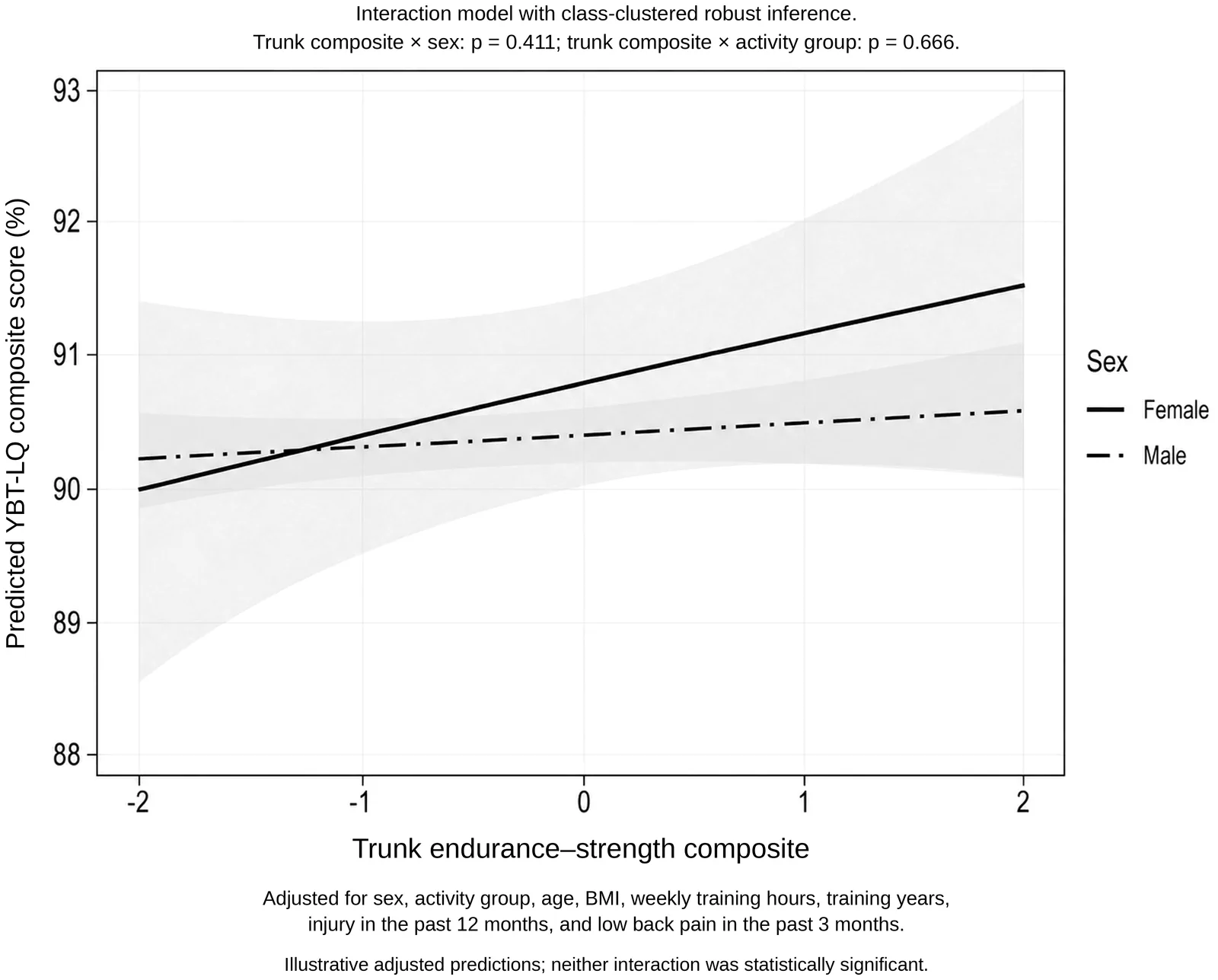
Adjusted predicted YBT-LQ composite score across levels of the trunk endurance–strength composite, by sex.

Model diagnostics are shown in **Supplementary Figure 1**. Sensitivity analyses are shown in **Supplementary Figure 2**. The positive associations of hip abductor strength and weight-bearing ankle dorsiflexion distance with YBT-LQ performance were consistent across the primary, outlier-excluded, and robust models. When the PCA-derived trunk-related factor was used instead of the trunk endurance–strength composite, the trunk-related estimate was attenuated, whereas the estimates for hip abductor strength and weight-bearing ankle dorsiflexion distance remained broadly similar.

## 4 Discussion

Dynamic balance performance is not determined by a single physical capacity; rather, it more likely reflects the coordinated contribution of the trunk, hip, and ankle during single-leg tasks. The central question of this study was not whether a core-related trunk endurance–strength composite was simply correlated with the YBT-LQ composite score, but whether that association retained stable independent explanatory value after adjustment for functional variables more closely aligned with the task itself. The answer provided by the present findings was relatively clear. The trunk endurance–strength composite was positively associated with the YBT-LQ composite score in the correlation analyses and in partially adjusted models, but its independent association was markedly attenuated after standardized hip abductor strength and standardized weight-bearing ankle dorsiflexion distance were introduced, whereas both variables remained robustly significant. In addition, the indirect pathway through hip abductor strength was statistically supported, whereas the indirect pathway through weight-bearing ankle dorsiflexion distance was not; neither the interaction terms nor most sensitivity analyses altered this overall pattern.

This pattern is more consistent with evidence conceptualizing dynamic balance as an integrated neuromuscular control task and does not fully support the view that a trunk endurance–strength composite alone is the most direct determinant of YBT-LQ performance. Training studies in young female athletes have shown that integrated neuromuscular training can improve Star Excursion Balance Test (SEBT) performance (Filipa et al., 2010). A systematic review of trunk training suggested that trunk muscle training can improve selected physical and sport-specific outcomes (Saeterbakken et al., 2022). However, another meta-analysis indicated that, in trained individuals, trunk muscle strength makes only a limited independent contribution to physical qualities and athletic performance and is better understood as one component within a broader capability set (Prieske et al., 2016). At the same time, studies in high-level athletes suggest that non-specific trunk stability tests may not capture the trunk adaptations that are truly relevant to sport tasks (Barbado et al., 2016). This helps explain why “core training may improve balance” and “the independent regression effect of core is unstable in cross-sectional data” can both be true: the former reflects global adaptations induced by training, whereas the latter reflects the residual independent information carried by the core indicator at a single time point after competition with variables more proximal and more distal to the task.

Compared with the trunk endurance–strength composite, hip abductor strength and weight-bearing ankle dorsiflexion distance showed more direct and more stable explanatory value in this study, a finding consistent with the task demands of the YBT-LQ. Research using the modified Star Excursion Balance Test has shown that dynamic balance performance is closely related to proximal lower-extremity control (Clagg et al., 2015). Biomechanical studies further indicate that individuals with insufficient hip abductor strength exhibit different ankle kinematics and neuromuscular activation patterns during single-leg balance tasks, suggesting that proximal control deficits can directly alter distal stabilization strategies (Lee and Powers, 2014). Previous evidence also supports the relevance of distal ankle mobility to dynamic balance performance (Hoch et al., 2011; Terada et al., 2014). From this perspective, the present study does not negate the importance of core stability; rather, it suggests that in a single-leg dynamic balance task such as the YBT-LQ composite score, more direct and more stable explanatory variables may arise from hip abductor function and weight-bearing ankle dorsiflexion distance. These findings are broadly consistent with the recent study by Olszewski et al. (2024), who reported that distal ankle mobility and hip abductor strength predicted YBT-LQ performance in healthy males. The similarity between their findings and the present results lies in the consistent importance of hip abductor strength and distal ankle function for interpreting YBT-LQ performance, while the present study specifically operationalized the ankle-related variable as weight-bearing ankle dorsiflexion distance. However, the two studies also differ in scope. Olszewski et al. focused on healthy males and direction-specific YBT-LQ performance, whereas the present study included physically active college students of both sexes and additionally examined whether the trunk endurance–strength composite retained independent explanatory value after hip and ankle factors were considered. Together, these findings enrich the current understanding of dynamic balance testing by suggesting that YBT-LQ performance is more strongly constrained by task-specific hip frontal-plane strength and weight-bearing ankle dorsiflexion distance than by broader or less task-specific physical measures alone. The supplementary functional measures provided contextual information on participants’ broader lower-extremity performance and motor-control profile.

The exploratory competing-pathway analyses further supported this hierarchical difference, but their implications should be interpreted cautiously. Because the data were cross-sectional, the statistically significant indirect association through hip abductor strength should be viewed as a statistical partial explanation or competing pathway rather than evidence of a temporally ordered physiological mechanism. Standardized weight-bearing ankle dorsiflexion distance showed a different pattern: it remained independently associated with the YBT-LQ composite score but did not form a significant indirect pathway. This suggests that weight-bearing ankle dorsiflexion distance functions more as a task constraint operating alongside trunk-related function than as the main transmission route of the trunk endurance–strength composite–YBT association in this study. In athletic populations, the link between balance capacity and performance is often more closely tied to the quality of task-specific motor control than to the isolated output of any single abstract physical construct (Hrysomallis, 2011). The absence of significant interaction terms makes this conclusion clearer: in this sample, there was insufficient evidence that sex or activity group systematically altered the association between core and dynamic balance, and therefore no additional narrative around moderation effects is warranted.

These findings have direct implications for the prioritization of assessment and intervention. If the goal is to explain or improve the YBT-LQ composite score, relying on a broad or nonspecific notion of “core stability” alone may be insufficient. The present findings suggest that the measured trunk endurance–strength composite should be interpreted together with hip abductor function and weight-bearing ankle dorsiflexion distance. A more reasonable approach is to first identify limitations in hip abductor function and weight-bearing ankle dorsiflexion distance, and then integrate trunk training into the lower-extremity control chain, rather than treating core training as a stand-alone module separate from the hip and ankle. Therapeutic exercise studies have shown clear differences across exercises in the levels of gluteus medius and gluteus maximus activation achieved (Distefano et al., 2009). This means that “training the core” is not the same as training the proximal musculature that genuinely contributes to single-leg control. For physically active college students or students majoring in sport-related disciplines, if the screening target is dynamic balance or lower-extremity control quality, hip abductor function and weight-bearing ankle dorsiflexion distance may warrant particular attention alongside trunk-related assessment; at the intervention level, the focus may not be simply increasing the volume of trunk training, but addressing trunk–pelvic control together with targeted management of hip and ankle function within a unified framework.

This study also has several limitations (Kibler et al., 2006): the core-related exposure was operationalized as a composite of trunk endurance and trunk strength rather than as a comprehensive measure of the multidimensional construct of core stability, and the cross-sectional design supports only associations and exploratory statistical decomposition. Therefore, the competing-pathway findings should not be interpreted as temporally ordered or causal mediation, and longitudinal or intervention studies are needed to determine whether changes in trunk-related capacity, hip abductor strength, or weight-bearing ankle dorsiflexion distance precede changes in YBT-LQ performance (Zemková and Zapletalová, 2022); the YBT-LQ composite score is an aggregated measure and may obscure directional asymmetries in the contributions of weight-bearing ankle dorsiflexion distance and hip control across different reach directions; and (Smith et al., 2015) the sample was drawn primarily from physically active or sport-major college students, so these findings are more applicable to young populations with similar training backgrounds; the relative importance of these variables may differ in sedentary populations, clinical populations, or athletes competing at a higher sport-specific level.

## 5 Conclusion

In physically active college students, although the trunk endurance–strength composite was positively associated with the YBT-LQ composite score, its independent association was markedly attenuated and became non-significant after simultaneous adjustment for hip abductor strength and weight-bearing ankle dorsiflexion distance. By contrast, hip abductor strength and weight-bearing ankle dorsiflexion distance remained stably and positively associated with dynamic balance, with hip abductor strength accounting for a substantial proportion of the statistical association between the trunk endurance–strength composite and dynamic balance.

## Data Availability

The original contributions presented in the study are included in the article/**Supplementary Material**. Further inquiries can be directed to the corresponding authors.

## Supplementary material

Supplementary Figure 1 Diagnostic plots for the primary regression model.

Supplementary Figure 2 Sensitivity analyses of key effect estimates across alternative models.

## References

[B1] Atkinson G. Reilly T. (1996). Circadian variation in sports performance. Sports Med. 21, 292–312. doi: 10.2165/00007256-199621040-00005 8726347

[B2] Barbado D. Barbado L. C. Elvira J. L. L. Dieën J. H. V. Vera-Garcia F. J. (2016). Sports-related testing protocols are required to reveal trunk stability adaptations in high-level athletes. Gait Posture 49, 90–96. doi: 10.1016/j.gaitpost.2016.06.027 27395448

[B3] Barrio E. D. Ramirez-Campillo R. Garcia de Alcaraz Serrano A. RaquelHernandez-García R. (2022). Effects of core training on dynamic balance stability: A systematic review and meta-analysis. J. Sports Sci. 40, 1815–1823. doi: 10.1080/02640414.2022.2110203 35976032

[B4] Clagg S. Paterno M. V. Hewett T. E. Schmitt L. C. (2015). Performance on the Modified Star Excursion Balance Test at the time of return to sport following anterior cruciate ligament reconstruction. J. Orthop. Sports Phys. Ther. 45, 444–452. doi: 10.2519/jospt.2015.5040 25899211

[B5] da Costa G. V. de Castro M. P. Sanchotene C. G. Ribeiro D. C. de Brito Fontana H. Ruschel C. (2021). Relationship between passive ankle dorsiflexion range, dynamic ankle dorsiflexion range and lower limb and trunk kinematics during the single-leg squat. Gait Posture 86, 106–111. doi: 10.1016/j.gaitpost.2021.03.015 33713896

[B6] De Los Ríos-Calonge J. Barbado D. Prat-Luri A. Juan-Recio C. Heredia-Elvar J. R. Elvira J. L. L. . (2024). Are trunk stability and endurance determinant factors for whole-body dynamic balance in physically active young males? A multidimensional analysis. Scand. J. Med. Sci. Sports 34, e14588. doi: 10.1111/sms.14588 38415784

[B7] Distefano L. J. Blackburn J. T. Marshall S. W. Padua D. A. (2009). Gluteal muscle activation during common therapeutic exercises. J. Orthop. Sports Phys. Ther. 39, 532–540. doi: 10.2519/jospt.2009.2796 19574661

[B8] Domínguez-Navarro F. Benitez-Martínez J. C. Ricart-Luna B. Cotolí-Suárez P. Blasco-Igual J. M. Casaña-Granell J. (2022). Impact of hip abductor and adductor strength on dynamic balance and ankle biomechanics in young elite female basketball players. Sci. Rep. 12, 3491. doi: 10.1038/s41598-022-07454-3 35241754 PMC8894415

[B9] Faul F. Erdfelder E. Buchner A. Lang A. G. (2009). Statistical power analyses using G*Power 3.1: Tests for correlation and regression analyses. Behav. Res. Methods 41, 1149–1160. doi: 10.3758/brm.41.4.1149 19897823

[B10] Filipa A. Byrnes R. Paterno M. V. Myer G. D. Hewett T. E. (2010). Neuromuscular training improves performance on the Star Excursion Balance Test in young female athletes. J. Orthop. Sports Phys. Ther. 40, 551–558. doi: 10.2519/jospt.2010.3325 20710094 PMC3439814

[B11] Hoch M. C. Staton G. S. McKeon P. O. (2011). Dorsiflexion range of motion significantly influences dynamic balance. J. Sci. Med. Sport 14, 90–92. doi: 10.1016/j.jsams.2010.08.001 20843744

[B12] Hooper T. L. James C. R. Brismée J. M. Rogers T. J. Gilbert K. K. Browne K. L. . (2016). Dynamic balance as measured by the Y-Balance Test is reduced in individuals with low back pain: A cross-sectional comparative study. Phys. Ther. Sport 22, 29–34. doi: 10.1016/j.ptsp.2016.04.006 27579805

[B13] Hrysomallis C. (2011). Balance ability and athletic performance. Sports Med. 41, 221–232. doi: 10.2165/11538560-000000000-00000 21395364

[B14] Kang M. H. Kim G. M. Kwon O. Y. Weon J. H. Oh J. S. An D. H. (2015). Relationship between the kinematics of the trunk and lower extremity and performance on the Y-Balance Test. Pm R. 7, 1152–1158. doi: 10.1016/j.pmrj.2015.05.004 25978949

[B15] Kibler W. B. Press J. Sciascia A. (2006). The role of core stability in athletic function. Sports Med. 36, 189–198. doi: 10.2165/00007256-200636030-00001 16526831

[B16] Lee S. P. Powers C. M. (2014). Individuals with diminished hip abductor muscle strength exhibit altered ankle biomechanics and neuromuscular activation during unipedal balance tasks. Gait Posture 39, 933–938. doi: 10.1016/j.gaitpost.2013.12.004 24373699

[B17] López-Valenciano A. Ayala F. De Ste Croix M. Barbado D. Vera-Garcia F. J. (2019). Different neuromuscular parameters influence dynamic balance in male and female football players. Knee Surg. Sports Traumatol. Arthrosc 27, 962–970. doi: 10.1007/s00167-018-5088-y 30088029

[B18] McGill S. M. Childs A. Liebenson C. (1999). Endurance times for low back stabilization exercises: Clinical targets for testing and training from a normal database. Arch. Phys. Med. Rehabil. 80, 941–944. doi: 10.1016/s0003-9993(99)90087-4 10453772

[B19] Nakagawa T. H. Petersen R. S. (2018). Relationship of hip and ankle range of motion, trunk muscle endurance with knee valgus and dynamic balance in males. Phys. Ther. Sport 34, 174–179. doi: 10.1016/j.ptsp.2018.10.006 30347312

[B20] Olszewski M. Zając B. Mika A. Golec J. (2024). Ankle dorsiflexion mobility and hip abductor strength can predict lower quarter Y-Balance Test performance in healthy males. J. Bodyw Mov. Ther. 38, 567–573. doi: 10.1016/j.jbmt.2024.03.053 38763610

[B21] Plisky P. J. Gorman P. P. Butler R. J. Kiesel K. B. Underwood F. B. Elkins B. (2009). The reliability of an instrumented device for measuring components of the Star Excursion Balance Test. N. Am. J. Sports Phys. Ther. 4, 92–99. PMC2953327 21509114

[B22] Powden C. J. Hoch J. M. Hoch M. C. (2015). Reliability and minimal detectable change of the weight-bearing lunge test: A systematic review. Man Ther. 20, 524–532. doi: 10.1016/j.math.2015.01.004 25704110

[B23] Preacher K. J. Hayes A. F. (2004). Spss and Sas procedures for estimating indirect effects in simple mediation models. Behav. Res. Methods Instrum. Comput. 36, 717–731. doi: 10.3758/bf03206553 15641418

[B24] Prieske O. Muehlbauer T. Granacher U. (2016). The role of trunk muscle strength for physical fitness and athletic performance in trained individuals: A systematic review and meta-analysis. Sports Med. 46, 401–419. doi: 10.1007/s40279-015-0426-4 26589515

[B25] Rabin A. Portnoy S. Kozol Z. (2016). The association of ankle dorsiflexion mobility with hip and knee kinematics during the lateral step-down test. J. Orthop. Sports Phys. Ther. 46, 1002–1009. doi: 10.2519/jospt.2016.6621 27686412

[B26] Saeterbakken A. H. Stien N. Andersen V. Scott S. Cumming K. T. Behm D. G. . (2022). The effects of trunk muscle training on physical fitness and sport-specific performance in young and adult athletes: A systematic review and meta-analysis. Sports Med. 52, 1599–1622. doi: 10.1007/s40279-021-01637-0 35061213 PMC9213339

[B27] Shaffer S. W. Teyhen D. S. Lorenson C. L. Warren R. L. Koreerat C. M. Straseske C. A. . (2013). Y-balance test: a reliability study involving multiple raters. Mil. Med. 178, 1264–1270. doi: 10.7205/MILMED-D-13-00222 24183777

[B28] Smith C. A. Chimera N. J. Warren M. (2015). Association of Y balance test reach asymmetry and injury in division I athletes. Med. Sci. Sports Exerc 47, 136–141. doi: 10.1249/mss.0000000000000380 24870573

[B29] Terada M. Harkey M. S. Wells A. M. Pietrosimone B. G. Gribble P. A. (2014). The influence of ankle dorsiflexion and self-reported patient outcomes on dynamic postural control in participants with chronic ankle instability. Gait Posture 40, 193–197. doi: 10.1016/j.gaitpost.2014.03.186 24768526

[B30] Thorborg K. Petersen J. Magnusson S. P. Hölmich P. (2010). Clinical assessment of hip strength using a hand-held dynamometer is reliable. Scand. J. Med. Sci. Sports 20, 493–501. doi: 10.1111/j.1600-0838.2009.00958.x 19558384

[B31] von Elm E. Altman D. G. Egger M. Pocock S. J. Gøtzsche P. C. Vandenbroucke J. P. (2008). The Strengthening the Reporting of Observational Studies in Epidemiology (Strobe) Statement: Guidelines for reporting observational studies. J. Clin. Epidemiol. 61, 344–349. doi: 10.1016/j.jclinepi.2007.11.008 18313558

[B32] Zemková E. Zapletalová L. (2022). The role of neuromuscular control of postural and core stability in functional movement and athlete performance. Front. Physiol. 13, 796097. doi: 10.3389/fphys.2022.796097 35283763 PMC8909639

